# Bacterial DnaK reduces the activity of anti-cancer drugs cisplatin and 5FU

**DOI:** 10.1186/s12967-024-05078-x

**Published:** 2024-03-12

**Authors:** Francesca Benedetti, Emmanuel F. Mongodin, Jonathan H. Badger, Arshi Munawwar, Ashley Cellini, Weirong Yuan, Giovannino Silvestri, Carl N. Kraus, Simone Marini, Chozha V. Rathinam, Marco Salemi, Hervé Tettelin, Robert C. Gallo, Davide Zella

**Affiliations:** 1 Institute of Human Virology, University of Maryland School of Medicine, Baltimore, MD USA; 2 Department of Biochemistry and Molecular Biology, University of Maryland School of Medicine, Baltimore, MD USA; 3 Department of Microbiology and Immunology, Institute for Genome Sciences, University of Maryland School of Medicine, Baltimore, MD 21201 USA; 4 Laboratory of Integrative Cancer Immunology, Center for Cancer Research, National Cancer Institute, National Institutes of Health, DHHS, Bethesda, MD USA; 5 Pathology Biorepository Shared Service, University of Maryland Greenebaum Comprehensive Cancer Center, Baltimore, MD 21201 USA; 6 Department of Medicine, University of Maryland School of Medicine, Baltimore, MD USA; 7 Aquestive, Warren, NJ USA; 8 Emerging Pathogens Institute, University of Florida, Gainesville, FL USA; 9 Department of Epidemiology, University of Florida, Gainesville, FL USA; 10 Department of Pathology, University of Florida, Gainesville, FL USA

**Keywords:** DnaK, Mycoplasma, Fusobacterium, Cisplatin, 5FU, TCGA, p53, Anti-cancer therapy

## Abstract

**Background:**

Chemotherapy is a primary treatment for cancer, but its efficacy is often limited by cancer-associated bacteria (CAB) that impair tumor suppressor functions. Our previous research found that *Mycoplasma fermentans* DnaK, a chaperone protein, impairs p53 activities, which are essential for most anti-cancer chemotherapeutic responses.

**Methods:**

To investigate the role of DnaK in chemotherapy, we treated cancer cell lines with *M. fermentans* DnaK and then with commonly used p53-dependent anti-cancer drugs (cisplatin and 5FU). We evaluated the cells’ survival in the presence or absence of a DnaK-binding peptide (ARV-1502). We also validated our findings using primary tumor cells from a novel DnaK knock-in mouse model. To provide a broader context for the clinical significance of these findings, we investigated human primary cancer sequencing datasets from The Cancer Genome Atlas (TCGA). We identified *F. nucleatum* as a CAB carrying DnaK with an amino acid composition highly similar to *M. fermentans* DnaK. Therefore, we investigated the effect of *F. nucleatum* DnaK on the anti-cancer activity of cisplatin and 5FU.

**Results:**

Our results show that both *M. fermentans* and *F. nucleatum* DnaKs reduce the effectiveness of cisplatin and 5FU. However, the use of ARV-1502 effectively restored the drugs' anti-cancer efficacy.

**Conclusions:**

Our findings offer a practical framework for designing and implementing novel personalized anti-cancer strategies by targeting specific bacterial DnaKs in patients with poor response to chemotherapy, underscoring the potential for microbiome-based personalized cancer therapies.

**Supplementary Information:**

The online version contains supplementary material available at 10.1186/s12967-024-05078-x.

## Background

The cancer-associated microbiota is one of the most significant components of the tumor microenvironment [1–3] with profound effects on anti-cancer drug response and toxicity [4], and a number of studies clearly show that the microbiota composition affects the effectiveness of chemotherapeutic drugs [5, 6]. In particular, cancer-associated bacteria (CAB) such as *Mycoplasma hyorhinis* [7–9] and *Fusobacterium nucleatum* [10–14] reduce the efficacy of certain anti-cancer drugs including gemcitabine, cisplatin and 5FU both in vivo and in vitro, though the molecular mechanism(s) involved are still largely unknown. Indeed, a complete map of the microbiota–host–drug interactome in cancer is lacking, mainly due to the difficulty in identifying the contribution of specific bacterial factors to both tumor development, progression and response to therapy. Understanding how the many players involved in this extremely complex biological system interrelate to prevent optimal drug response would pave the way for the development of effective anti-cancer strategies.

Colorectal and gastric cancers are among the leading cause of tumor-related mortality, both in the United States and worldwide [15]. For the treatment of both cancers is widely used a combination therapy regimen comprising platinum-based molecules like cisplatin and/or the anti-metabolite 5-Fluorouracil (5FU) [16–18]. Administration of either molecule results in DNA damage and RNA synthesis inhibition, leading to cell death through a series of cellular events not completely fully understood, but which mainly involves p53 activation [19–29]. Indeed, most anti-cancer drugs rely on the induction or blockage of DNA repair, with consequent activation of p53 followed by apoptosis to exert their function.

We previously showed that Mycoplasma DnaK, a chaperone protein belonging to the Hsp70 family, binds to USP10 (ubiquitin carboxyl-terminal hydrolase 10), a regulator of p53 stability [30]. This binding in turn reduces the activities of p53 [30, 31], an essential transcription factor that promotes cell cycle blockage and apoptosis in the presence of extensive DNA damage [32]. Of note, DnaK/HSP70 [33] may be released by the bacteria [34, 35] and then taken up by uninfected cells [30, 31, 36] or directly translocated into the eukaryotic cells upon attachment or invasion [37–39]. All these data would clearly establish DnaK as a constituent of the tumor microenvironment, with the likely ability to affect the effectiveness of some anti-cancer drugs.

Here we investigated the effect of *M. fermentans* DnaK on the anti-cancer activity of cisplatin and 5FU. We showed that DnaK exogenously added to human cancer cell lines greatly reduces the efficacy of both anti-cancer drugs, while a DnaK-binding peptide completely restored their activity. Next, we confirmed these data in primary tumor cells from a knock-in mouse model constitutively expressing DnaK generated in our laboratory. By mining human primary cancer sequencing datasets from The Cancer Genome Atlas (TCGA) we then detected other CAB carrying DnaKs with highly similar amino acid composition. Among them, we identified *F. nucleatum* and demonstrated that also its DnaK can inhibit the anti-cancer efficacy of cisplatin and 5FU when exogenously added to cancer cell lines. In conclusion, we highlight a new mechanism whereby bacteria hamper anti-cancer effects of widely used chemotherapeutic agents. Current anti-cancer drugs regimens should consider these data to design better personalized treatments in cancer patients when planning treatment protocols or when considering causes of failing regimens.

## Materials and methods

### Cell lines

A human colorectal carcinoma cell line (HCT116, CCL-247) and a gastric adenocarcinoma cell line (AGS, CRL-1739) used in the experiments were all from American Type Culture Collection (ATCC). The cells were cultured in a humidified incubator at 37 °C in 5% CO_2_ in McCoy medium (HCT116) or F-12K medium (Kaighn’s Modification of Ham’s F-12 medium) (AGS), all containing 10% fetal bovine serum (FBS), 100 U/mL penicillin, 100 U/ml streptomycin and 290 µg/mL l-glutamine (all from ThermoFisher Scientific, Waltham, MA, USA). The identity of cell lines was confirmed by short tandem repeat (STR) profiling. The analysis has been conducted at Labcorp (Burlington, NC), followed by the comparison with STR profiles of known references. We used CLASTR 1.4.4, a Cellosaurus STR similarity search tool (http://web.expasy.org/cellosaurus/), to authenticate the cells used in our experiments [40, 41]. Our parameters have been the following: “Tanabe” as scoring algorithm and 70% as score filter.

### Expression and purification of *Mycoplasma fermentans* (eM-DnaK) and *Fusobacterium nucleatum* (eF-DnaKs) exogenous proteins

Recombinant exogenous DnaKs-V5 used in this study were obtained as previously described [30, 42]. Briefly, both eM-DnaK and eF-DnaK sequences were inserted into a cloning vector for the expression of the protein fused to a V5-tag peptide. They were then subcultured into TB/LB with Kanamycin, followed by fractionation and purification (Biomatik USA, Wilmington, DE). After this step, the proteins were extensively dialyzed against PBS 1× (pH 7.4), and Coomassie blue-stained SDS-PAGE (> 85%) was used to determine their purity. The proteins were then aliquoted to avoid frequent freeze-thaws and kept at − 80 °C after reconstitution.

### DnaK knock-in mice and isolation of primary cells

Transgenic animals were generated in collaboration with Taconic Biosciences (Rensselaer, NY). Briefly, the “CAG-Kozak-DnaK-V5 tag-TAGTAG-polyA” cassette was cloned into intron 1 of ROSA26 in reverse orientation. The V5 tag was added to conveniently detect DnaK, which was inserted in the ROSA26 locus by using the CRISPR/Cas9-mediated genome editing technology. All animal experiments were performed in accordance with relevant guidelines and regulations and were approved by the University of Maryland School of Medicine Institutional Animal Care and Use Committee (IACUC). Mice were monitored daily and when a spontaneous solid tumor mass was detected, the mouse was euthanized, and the mass carefully removed. A portion of the tumor mass was placed in formalin and then sent to the American Histolabs (Gaithersburg, MD) for the paraffin embedding and the Hematoxylin and Eosin staining of the slides. Pictures of the slides has been taken using an Olympus BX43 microscope (DP72 camera) and the CellSens Standard software (Olympus). The rest of the cancer cells were separated in a single-cell suspension from the intact tissue by mechanical force and then cultured under normal culturing conditions in RPMI + 10% FBS (37℃, 5% CO_2_) and partially frozen at − 80 °C.

### Treatments with anti-cancer drugs (Cisplatin and 5FU) and ARV-1502 peptide

To determine the effects of eM-DnaK and eF-DnaKs on HCT116 and AGS cells lines treated with different anti-cancer drugs, cells were plated 200,000 cells/well in 6-wells plates. After 24 h, both eM-DnaK and eF-DnaK were added to the cultures at a concentration of 10 µg/mL. After 24 h, anti-cancer drugs (cisplatin 25 µM, 5FU 75 µM) were added to the cells (both treated and not treated with DnaKs). We selected these concentrations of platinum-based drugs or 5FU to decrease the number of viable cells by at least 50%. Cisplatin was from Selleckchem (Houston, TX), while 5FU was from Sigma-Aldrich (St. Louis, MO). Parallel cultures of untreated cells were the negative controls. Also, parallel treatments of DMF (control for cisplatin treatment, dissolved in DMF following manufacturer’s instructions) and DMSO (control for 5FU treatment, dissolved in DMSO based on manufacturer’s instructions) have been used as negative controls. Cells treated with DMF or DMSO did not show increased cell death and their proliferation rate remain normal. Thus, we used untreated cells as negative control. After 48 h of treatment with the anti-cancer drugs, cell monolayers were washed in PBS, trypsinized and cell viability was measured using the trypan blue assay. The trypan blue exclusion assay allows for a direct identification and enumeration of live (unstained) and dead (blue) cells in the given population.

To verify that bacterial DnaK was responsible for reduction in platinum-based drugs and 5FU anti-cancer-activities, we used a peptide (ARV-1502, optimized from pyrrhocoricin and drosocin) which binds to *Escherichia coli* DnaK substrate-binding domain and to decreases its ATPase activity [43–46]. More in detail, we pre-treated both exogenous DnaKs with ARV-1502 (25 µg/mL) before adding them to the culture of HCT116 or AGS cells. After 3 h of incubation the complex was added to the culture. After 24 h the cells were treated with the anti-cancer drugs and then subjected to count with trypan blue as previously described. Parallel cultures of cells treated with the drugs and DnaKs not complexed with ARV-1502 were used as control.

We followed the same experimental procedures described above for the treatment of the primary murine cancer cells (ex vivo experiments). In particular, primary cancer cells from DnaK knock-in mice were treated with the same concentrations of anti-cancer drugs (cisplatin and 5FU) and with the same concentration of DnaK inhibitor (ARV-1502), for the duration of the experiments. As before with the exogenous DnaKs, the primary cancer cells have been pretreated with ARV-1502 for 24 h before adding the anti-cancer drugs.

Statistical differences in the means were tested using Student’s t test. All statistical tests were two-sided.

### Western blotting

Western blot was performed to verify the expression of DnaK in the mouse primary cancer cells, in the internalization of the exogenous DnaKs and to validate DnaK binding with ARV-1502. HCT116 cells were treated with eM-DnaK, with or without ARV-1502, with or without Cisplatin, as described in the previous section of Methods. eM-DnaK was added to the cells at a concentration of 10 µg/mL. After 72 h since eM-DnaK treatment, cell monolayers were washed in cold PBS, trypsinized and resuspended in RIPA lysis buffer (Sigma-Aldrich, St. Louis, MO) in the presence of protease inhibitors (Sigma-Aldrich, St. Louis, MO). The protein concentration was measured by the Bradford assay (Bio-Rad Laboratories, Hercules, CA). Thirty micrograms of protein were resolved by SDS/PAGE, transferred to a polyvinylidene difluoride (PVDF) membrane using trans-blot turbo transfer system (Bio-Rad Laboratories, Hercules, CA), blocked in 5% nonfat dried milk in Tris-Buffered Saline (TBS) and probed overnight with either a mouse mAb against the V5 tag (#R960-25, Thermo Fisher Scientific, Walthman, MA) to detect the presence of eM-DnaK or a mouse mAb against β-actin (8H10D10) (#3700, Cell Signaling Technology, Danvers, MA). Blots were then incubated with a secondary HRP-conjugated antibody (Cell Signaling Technology, Danvers, MA) and developed using an ECL chemiluminescent substrate kit (Genesee Scientific, San Diego, CA). They were then exposed and acquired using the ChemiDoc MP digital image system (Bio-Rad Laboratories, Hercules, CA).

The untreated primary cancer cells underwent the same procedures. Briefly, the total proteins were extracted and quantified, and after running and blotting, the membranes were probed overnight with either a primary rabbit mAb antibody against the V5 tag (#ab182008, Abcam, Cambridge, UK) to detect the presence of DnaK-V5, or a rabbit mAb against GAPDH (14c10) (#2118, Cell Signaling Technology, Danvers) used as housekeeping. We resolved in the same gel eM-DnaK protein as positive control and proteins obtained from a spleen of a DnaK^−/−^ animal as negative control.

### Surface plasmon resonance (SPR) binding analysis of DnaK-ARV-1502

Surface plasmon resonance (SPR) binding studies of DnaK and ARV-1502 were performed at 25 °C on a BIAcore T100 System (BIAcore, Inc., Piscataway, NY). We used as assay buffer HBS-EP, containing 10 mM HEPES, 150 mM NaCl, 0.05% surfactant P20, pH 7.4, 3 mM EDTA. DnaK (2274.9 RUs) was immobilized on CM5 sensor chips using the amine-coupling chemistry recommended by the manufacturer. Analytes were introduced into the flow cells at 35 μL/min in the running buffer. Association and dissociation were assessed for 250 s and 600 s. Resonance signals were corrected for nonspecific binding by subtracting the background of the control flow-cell. After each analysis, the sensor chip surfaces were regenerated with 10 mM glycine solution (pH 2.0) with MgCl 1 M and equilibrated with the buffer before the next injection.

### TCGA analysis

The presence and distribution of bacteria within human cancer tissues, especially *Mycoplasma* and *Fusobacterium*, was assessed through data mining of human primary cancer sequencing datasets from The Cancer Genome Atlas (TCGA). TCGA hosts human genomic and transcriptomic sequencing data sets from a large number of human cancer tissues [47], where bacterial sequences can also be retrieved and analyzed to characterize CAB.

RNA-Seq sequences from a total of 10,293 samples spanning 33 different cancer types were initially retrieved from TCGA (version 9.0), after which analyses were focused only on primary tumor samples and solid tissue normal samples. As such, samples from the following cancers were removed from the analyses: acute myeloid leukemia, lymphoid neoplasm diffuse large B-cell lymphoma, mesothelioma, skin cutaneous melanoma, cholangiocarcinoma, testicular germ cell tumors, as well as metastatic, additional metastatic and “additional—new primary” samples. After sample filtering, the final dataset analyzed was comprised of 9505 primary solid tumor and solid tissue normal samples distributed across 27 cancer types. To note, some solid tissue normal samples (uveal melanoma, uterine carcinosarcoma, ovarian serous cystadenocarcinoma, glioblastoma multiforme, brain lower grade glioma, adrenocortical carcinoma) were missing in the dataset. Sequences were downloaded in BAM alignment format from TCGA, and reads which were indicated in the alignment as mapping to the human genome were discarded, since we needed to retain only the potential microbial sequences. To distinguish microbial sequences from other sequences that did not map to the human genome (such as sequencing artifacts or mutations/rearrangements within tumors) we first screened the sequences with a Hidden Markov Model (HMM) [48] created from the SILVA Release 132 alignment [49] and then taxonomically classified the sequences using Kraken 2 [50] with a database also based on SILVA 132. The resulting 16S sequence dataset was taxonomically assigned to a total of 9510 taxa at 7 different taxonomic levels (from phylum to species) and count tables were generated for data visualization and analyses in R. Because of the wide variations in the number of reads sequenced across all samples (min: 49,637,151 sequencing reads; max: 516,415,337 sequencing reads; Additional file 3: Fig. S3A, B), 16S counts were then normalized in each sample by computing a scaling factor based on the number of reads in a sample divided by the number of reads in the smallest sample.

### Specimen collection and detection of bacterial DnaK using qPCR

Frozen biopsies of cancers tissues already identified in the Pathology Biorepository Shared Service (PBSS) core of the Greenebaum Comprehensive Cancer Center at the University of Maryland (GCCC-UM) were collected and stored in deep freezer (− 80 °C). This retrospective study was approved by the Institutional Review Board at University of Maryland, Baltimore (approval number: HP-00040021). All methods were performed following the relevant guidelines and regulations. Documented informed consent was obtained from each study participant. Patient demographics and clinical characteristics were investigated by reviewing the medical records and interviews. Minimal associated clinic-pathologic data to include tumor histologic type, treatment status (treatment naïve vs. post neoadjuvant treatment), treatment regimen, and an assessment of patient treatment responses was collated. Since the samples were obtained to validate a proof of concept, inclusion criteria, such as sex, age or weight, randomization, blinding, power analysis, were considered irrelevant for the study’s objectives. Total DNA was extracted from tissues using the DNeasy Blood & Tissue Kits (QIAGEN, Hilden, Germany) according to the manufacturer’s instructions. DNA concentration and purity were recorded using a NanoDrop spectrophotometer (NanoDrop Technologies, Wilmington, DE). *Mycoplasma* and *Fusobacterium* DnaK genes were detected and amplified by qPCR using the following primers and probes:

- eM-DnaK: F primer: CAA TGC ACA ACG TGA AGC CA; R primer: AAG CAG CAG CAG TAG GTT CG; probe: 5 6-FAM/AT CGC AGG T/ZEN/A AAA TTG CAG G/3IABkFQ/;
- eF-DnaK*:* F primer: CAA CAC AAG GAC CTA CAA AAA C; R primer: CGC AAC AAC TTC ATC AGG G; probe:/56-FAM/AA ATC TTA C/ZEN/T TGT TGG AGG TTC TAC AAG AAT ACC A/3IABkFQ/.

Briefly, amplifications were performed in 20 μL reaction mixture containing 1× SsoAdvanced Universal Probes Supermix (Bio-Rad Laboratories, Hercules, CA), each primer at 300 nM, probe at 200 nM and 50 ng of total DNA. Reference standard curves were generated using serially diluted plasmids containing the target DnaK gene. Aliquots were prepared once by dilution of DNA in distilled water and were stored at − 20 °C. Water and aliquots of total DNA from HCT116 and AGS cells *Mycoplasma* and *Fusobacterium*-free were included for each of the amplifications as negative controls.

Following activation of DNA polymerase at 94 °C for 30 s, 40 cycles of amplification (denaturation step, 95 °C for 15 s; annealing-extension step, 60 °C for 30 s) were performed with CFX384 Real-Time PCR System (Bio-Rad Laboratories, Hercules, CA). An accurate analysis of the melting temperature curve of the generated amplicons was conducted for of the amplifications to rule out any non-specific interference.

### Generation of data for DnaK domains’ comparison

We started from the *Mycoplasma fermentans* (MF-I1—ATFG00000000) [30] template sequence for DnaK. The template sequence is reported in Additional file 5: Table S2. We extracted three domains for DnaK. The exact positions of the regions of interested are reported, separated by semicolons, in the respective sequence headers. Domain 1 (NDB) extended within aa1-392, domain 2 (SBD) within aa392-507, and finally domain 3 (α-helical domain) within aa508-638, as described [51]. We downloaded 22,155 DnaK bacterial proteins from NCBI (query: DnaK_hsp70). We then aligned each fragment of each template against ncbi-blast-2.9.0 against the target downloaded proteins. Blast results were filtered to keep only matches > 70% of the length of the query, after which the genus information of the matches was parsed to generate the distribution plots in Fig. 5.

## Results

### Exogenous Mycoplasma DnaK reduces the activity of cisplatin and 5-fluorouracil in human cancer cell lines

To test the hypothesis that exogenously Mycoplasma DnaK added to cells treated with cisplatin or 5FU could reduce their anti-cancer effect, we designed an in vitro assay based on the colorectal carcinoma cell line HCT116 and the gastric carcinoma cell line AGS. This assay also uses purified exogenous *M. fermentans* DnaK (eM-DnaK) recapitulating the conditions whereby cancer cells would take up [30, 36] the bacterial protein released in the surrounding tumor microenvironment [34, 37, 52]. This in turn allowed us to study DnaK’s effect on cell viability in the presence of the anti-cancer drugs.

**Fig. 1:**
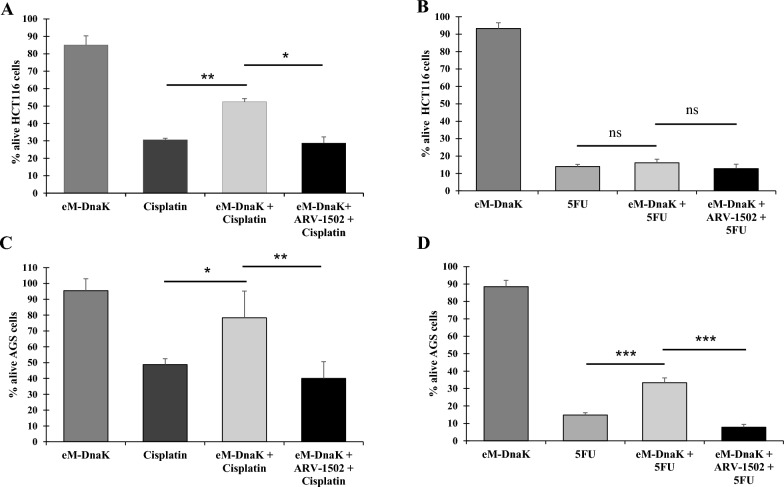
Effect of eM-DnaK and ARV-1502 on viability of HCT116 and AGS cell lines treated with cisplatin and 5FU. Cisplatin 25 µM (**A**–**C**) and 5FU 75 µM (**B**–**D**) were added to each well with the indicated cell line alone or in combination with eM-DnaK. Parallel wells of untreated cells were used as negative control. Cell viability was assessed by using the trypan blue assay. Percentage of alive cells for each treatment are calculated as percentage using the untreated cell as reference. Results are representative of 3 independent experiments for each treatment. Statistical differences were tested using Student’s t test. All statistical tests were two sided. ***p < 0.001, **p < 0.01, *p < 0.05, ns = not significant. Treatment with ARV-1502 alone showed on average 5–8% reduction in cell viability (data not shown to maintain a clearer visibility of the results)

When HCT116 cells were treated with cisplatin in the presence of eM-DnaK, the anti-cancer effect of the drug was blunted, and viability greatly increased from 31 to 53% (Fig. 1A). On the other hand, the presence of eM-DnaK did not have a statistically significant effect on the anti-cancer action of 5FU, and viability increased only from 14 to 16% (Fig. 1B).

When AGS cells were treated with cisplatin in the presence of eM-DnaK, the anti-cancer effect of the drug was also reduced, leading to an increase in cell viability from 40 to 57% (Fig. 1C). Similarly, a reduction of anti-cancer activity was also observed following treatment with 5FU in the presence of eM-DnaK, with viability increasing from 15 to 33% (Fig. 1D).

### A specific DnaK-binding peptide restores the drugs’ anti-cancer activities

To confirm that bacterial DnaK is responsible for reduction in cisplatin and 5FU anti-cancer-activities, and to provide proof of concept for therapeutic intervention, we used ARV-1502, a peptide optimized from pyrrhocoricin and drosocin, which has been previously demonstrated to bind the *Escherichia coli* DnaK substrate-binding domain and to reduce its ATPase activity, without interacting with human Hsp70 [43, 53, 54]. We first show that the peptide is also able to bind eM-DnaK (Additional file 1: Fig. S1A) and that ARV-1502 binding to DnaK is not preventing the exogenous protein entry into the cells (Additional file 1: Fig. S1B). Then, we proceeded to analyze the effects of ARV-1502 in HCT116 and AGS cells treated with cisplatin and 5FU in the presence of eM-DnaK. In all samples we observed a restoration of the original anti-cancer activity of each drug, indicating that the inhibitory effect of DnaKs was being reversed (Fig. 1A–D).

### ARV-1502 increases the activity of anti-cancer drugs in mouse primary cancer cells expressing Mycoplasma DnaK protein

**Fig. 2:**
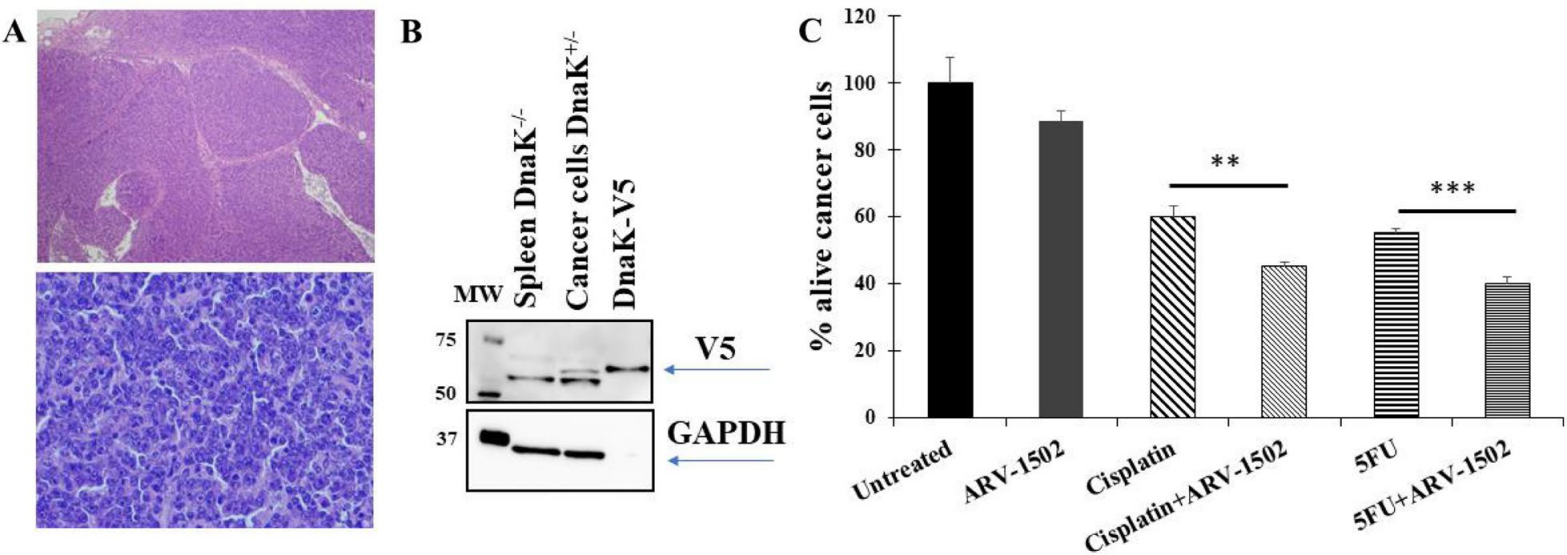
ARV-1502 increases anti-cancer activity of cisplatin and 5FU in cells from a murine primary cancer constitutively expressing DnaK. **A** Hematoxylin and Eosin (H&E) staining of a spontaneous mass removed from the abdomen of a DnaK positive mouse. The normal architecture is effaced by unencapsulated, poorly demarcated, densely cellular neoplasm composed of round cells arranged in sheets. Neoplastic cells have variably distinct cell borders, a scant amount of eosinophilic cytoplasm, a round, occasionally indented nucleus with finely stippled chromatin and one variably prominent nucleolus. Anisocytosis and anisokaryosis are moderate, and mitotic count is up to 7 in a single high-power field (2.32 mm^2^). These findings are consistent with a round cell neoplasia. The images of the section were taken at 4× (top) and 40× (bottom). **B** Western Blot analysis confirms expression of DnaK-V5 in the murine primary cancer cells isolated from the spontaneous tumor. Both eM-DnaK and DnaK expressed in cancer cells were fused to a V5 peptide sequence for convenient detection. eM-DnaK has been used as positive control for antibody detection. Cells isolated from a spleen of a DnaK^−/−^ mouse were used as negative control. Upper part: membrane probed with anti-V5 antibody recognizing DnaK-V5. Lower part: membrane probed with an anti-GAPDH antibody. Markers specifying the molecular weight (MW) are indicated. **C** Viability assay of primary murine cancer cells treated with anti-cancer drugs with or without ARV-1502. Cells from the spontaneous tumor mass (round cell neoplasia) detected in a DnaK positive mouse were isolated and then treated with the anti-cancer drugs, cisplatin (25 µM) or 5FU (75 µM). In parallel, the cells were also treated with ARV-1502. We assessed cell viability by using the trypan blue assay. Percentage of alive cells for each treatment are calculated as percentage using untreated cell as reference. The results are representative of two independent experiments using primary cells from two different spontaneous tumors. Statistical differences were tested using Student’s t test. All statistical tests were two sided. ***p < 0.001, **p < 0.01

To validate ex vivo our previous data in cell lines, we next used primary cancer cells derived from a spontaneous solid tumor mass (round cell neoplasia) retrieved from the abdomen of a DnaK positive knock-in mice generated in our Laboratory (Fig. 2A). These cells constitutively express DnaK mimicking an in vivo situation whereby the cells would be infected by *M. fermentans* expressing and secreting DnaK inside the cell’s compartments [39] (Fig. 2B). Treatment of the cancer cells with cisplatin or 5FU reduced their viability to 60% and 55%, respectively (Fig. 2C). The treatment with the DnaK inhibitor ARV-1502 alone had a slight inhibitory effect (11%), resulting in reduced cells viability to 89% compared to the untreated cells. When the primary cancer cells were treated with the DnaK inhibitor ARV-1502 we observed cell viability further reduced to 45% for cisplatin and to 40% for the 5FU (Fig. 2C), which amounts to a 25% improved anti-cancer effect for both drugs. These data indicate that inhibiting DnaK activity re-established the activity of anti-cancer drugs in the primary cancer cells, confirming the data obtained in the cancer cell lines.

### Identification of CAB with amino acid composition similar to Mycoplasma DnaK

**Fig. 3:**
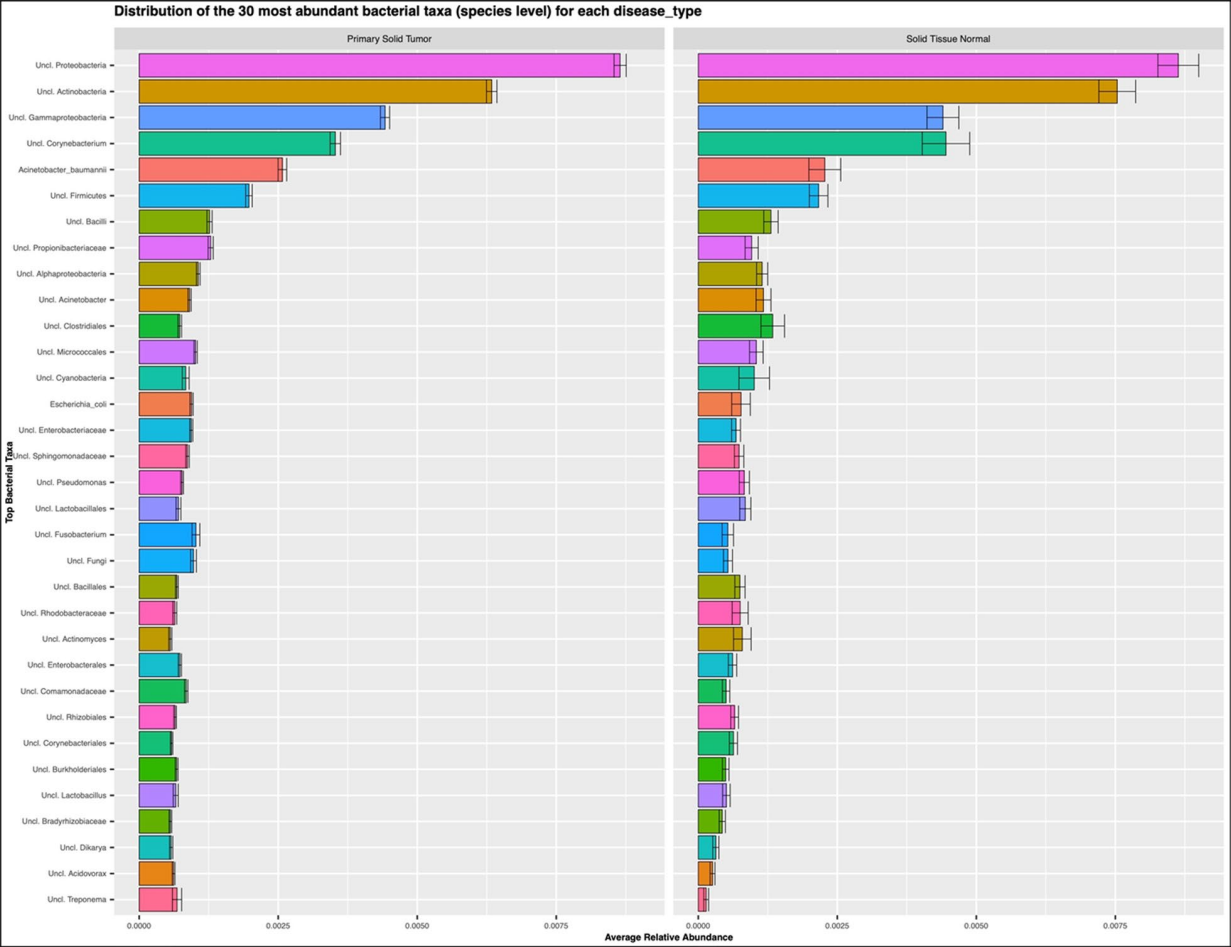
Distribution of the 30 most abundant bacterial taxa (species level) in both primary solid tumors and normal solid tissues

**Fig. 4:**
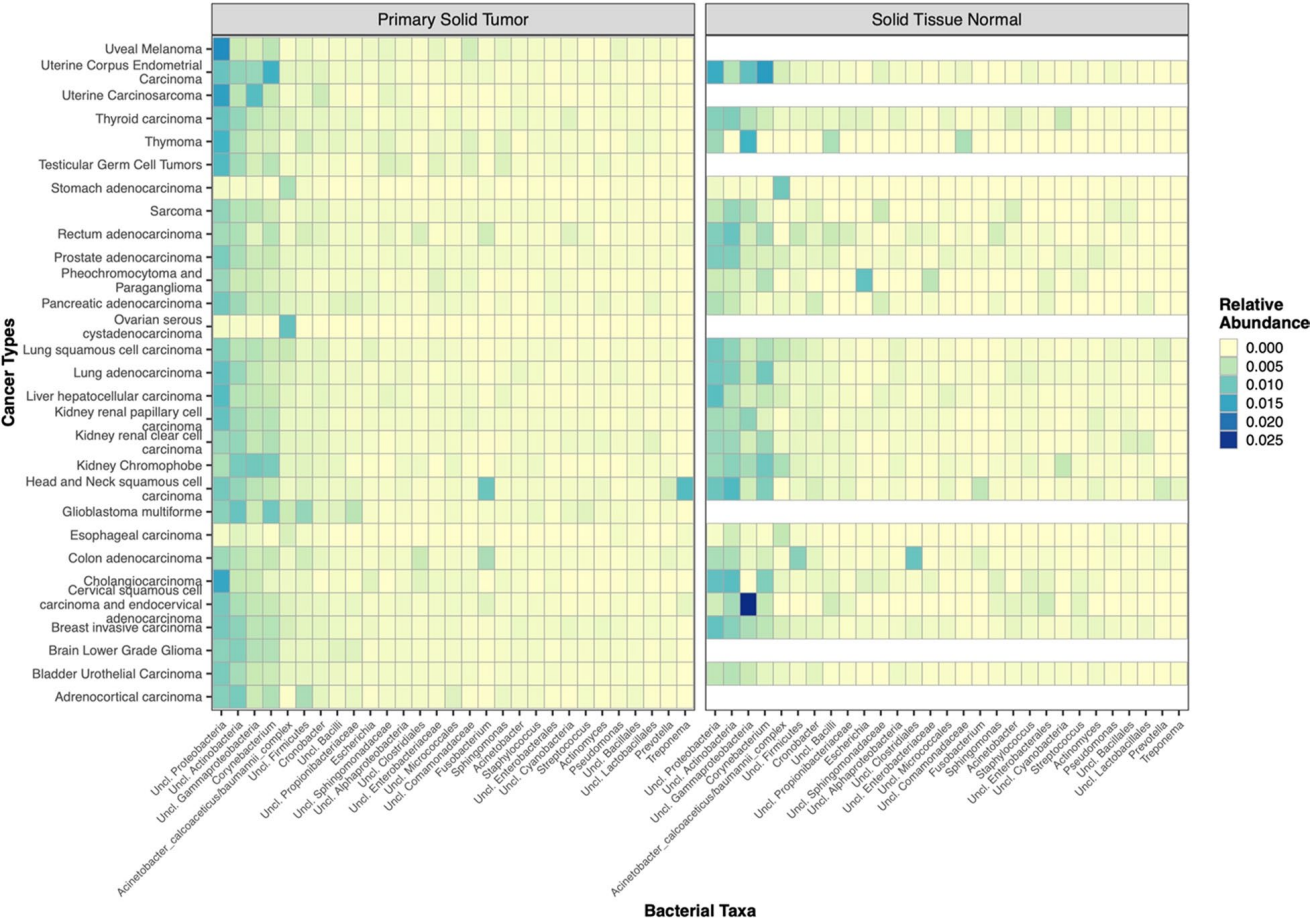
Heatmap displaying relative abundance values of bacteria identified in primary solid tumor and normal solid tissue

The Cancer Genome Atlas provides a comprehensive dataset of nucleic acid sequences, both DNA and mRNA from a number of cancer tissues [2, 47, 55]. We reasoned that bacterial sequences could be retrieved from this dataset and used to evaluate the composition of the cancer-associated microbiota and the expression of different bacterial genes, after removal of all the eukaryotic sequences from the mRNA dataset (see also “Materials and methods”). Using the 16S rRNA gene sequences identified in the TCGA data set, we characterized bacterial taxa profiles from 9505 primary solid tumor and solid tissue normal samples distributed across 27 cancer types (Additional file 2: Fig. S2). On average, 263,379 16S rRNA reads were identified in each cancer type with a wide variability across cancer types (from a min of 79 16S sequences in kidney renal carcinomas to 38,415,049 16S sequences in ovarian serous cystadenocarcinoma; Additional file 2: Fig. S2 bottom panel) which was concomitant with the wide range in the sequencing data set size for each sample. Overall, the top 5 bacterial taxa detected across all samples (both primary solid tumor and solid tissue normal) were *Proteobacteria*, *Actinobacteria, Gammaproteobacteria*, *Corynebacterium* (all four taxa unclassified at the genus level) and *Acinetobacter baumanii* bacteria (Fig. 3). The general bacterial profiles, obtained by aggregating samples across all cancers, seemed similar when comparing primary solid tumor to solid tissue normal samples (Fig. 4). Nonetheless, previous studies have shown that bacterial biomarkers of cancer can be identified for specific cancer types [2, 56, 57] and that specific bacteria compose the tumor microbiome [1], hinting at potential role of specific bacteria in certain cancers [58].

**Fig. 5:**
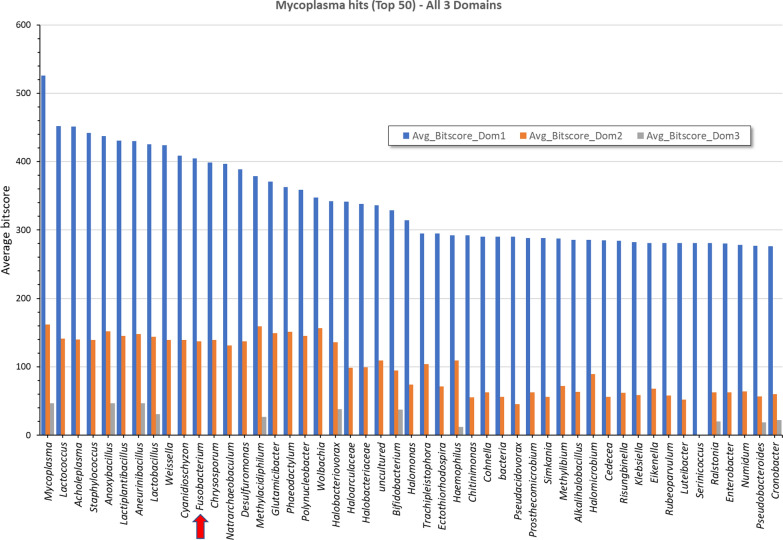
Genus distribution of the top 50 Blast hits for the 3 DnaK domains of Mycoplasma. Domain 1 (NDB) aa1-392, domain 2 (SBD) aa392-507 and domain 3 (α-helical domain) aa508-638, as described [51]. *Fusobacterium* is indicated by an arrow

To identify other DnaKs that could have the same inhibitory effect on anticancer drugs, we searched for bacterial DnaKs with amino acid composition similar to the different domains of *Mycoplasma* DnaK, which consists of an N-terminal ATPase domain of about 45 kDa (NBD, nucleotide binding domain) and a C-terminal substrate of about 25 kDa (SDB, substrate binding domain). The latter is further subdivided into a *β*-sandwich subdomain of about 15 kDa and a C-terminal *α*-helical subdomain of 10 kDa [51]. We obtained a list of the first 50 bacterial hits by average bitscore for each domain of Mycoplasma DnaK, which is presented in Fig. 5. Among the identified bacteria, *Fusobacterium* DnaK exhibited a high degree of similarity with *M. fermentans* DnaK. These results extend and confirm at the domain level our previous phylogenetic amino acid analysis [30]. We note that several reports indicate that *F. nucleatum* is commonly associated with gastrointestinal cancer [59–61] and progression with cancer and resistance to anti-cancer therapy [10–12].

### The activity of anti-cancer drugs is reduced by *Fusobacterium nucleatum* DnaK and restored by ARV-1502

**Fig. 6:**
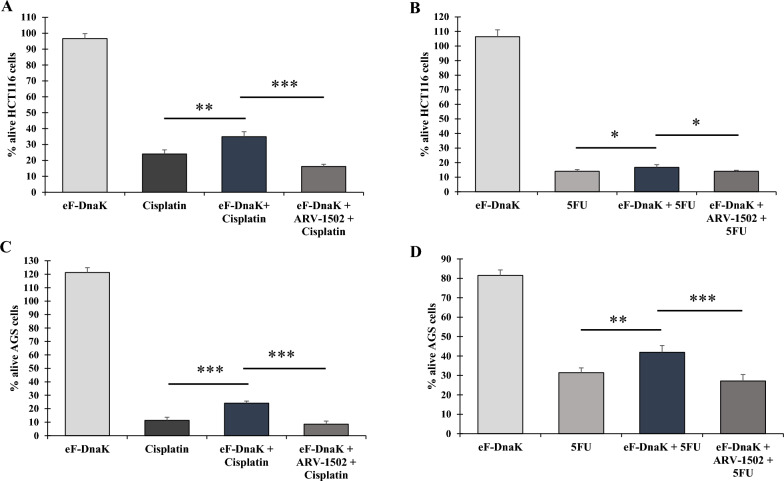
Effect of eF-DnaK and ARV-1502 on viability of HCT116 and AGS cell line treated with cisplatin and 5FU. Cisplatin 25 µM (**A**–**C**) and 5FU 75 µM (**B**–**D**) were added to each well with the indicated cell line alone or in combination with eF-DnaK. Parallel wells of untreated cells were used as negative control. Cell viability was assessed by using the trypan blue assay. Percentage of alive cells for each treatment are calculated as percentage using the untreated cell as reference. Results are representative of 3 independent experiments for each treatment. Statistical differences were tested using Student’s t test. All statistical tests were two sided. *p < 0.05, **p < 0.01, ***p < 0.001. Treatment with ARV-1502 alone showed on average 5–8% reduction in cell viability (data not shown to maintain a clearer visibility of the results)

Given the similarity in amino acid composition between *Mycoplasma* and *Fusobacterium* DnaKs, we asked whether exogenously added *F. nucleatum* DnaK (eF-DnaK) could reduce the activity of cisplatin and 5FU. When cisplatin was used for the treatment of HCT116 cells in the presence of eF-DnaK, the anti-cancer effect of the drug was blunted and viability increased from 24 to 35% (Fig. 6A). Also, the anti-cancer-effect of 5FU was reduced in the presence of eF-DnaK, and viability increased from 14 to 17% (Fig. 6B).

When AGS cells were treated with cisplatin in the presence of eF-DnaK, the anti-cancer effect was reduced and viability increased from 11 to 24% (Fig. 6C). Treatment with 5FU in the presence of eF-DnaK also reduced its anti-cancer effect, and viability increased from 31 to 42% (Fig. 6D).

Similarly to what observed with eM-DnaK, treatments with ARV-1502 were able to restore the drugs’ anti-cancer activity in the presence of eF-DnaK (Fig. 6A–D, and also cf. Fig. 1A–D).

**Fig. 7:**
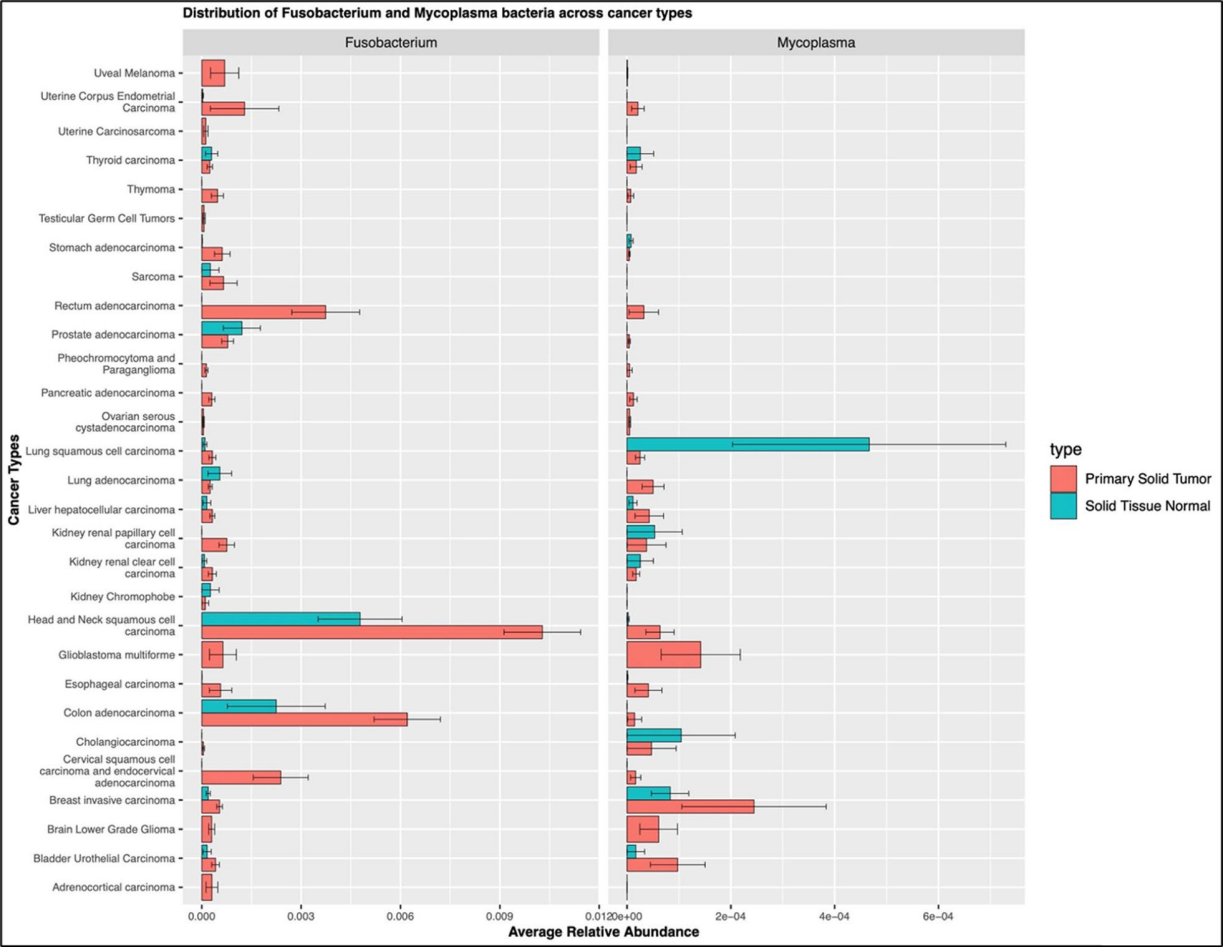
Distribution of *Fusobacterium* and *Mycoplasma* bacteria across cancer types. The distribution of both Fusobacterium and Mycoplasma bacteria was determined in the different cancer samples belonging to the TCGA data set, as described in “Materials and methods”. The average relative abundance and distribution of each bacterium in primary solid tumors and solid tissue normal are indicated

Next, we performed an analysis of bacteria associated with individual cancer types from the TCGA dataset. In some cases, we highlighted clear differences comparing solid tumor tissues to normal samples (Fig. 7). *Fusobacterium* is more frequently present in the primary solid tumor across all samples compared to normal tissues, except for a few types of cancers (namely, prostate adenocarcinoma, lung adenocarcinoma, and kidney chromophobe) where it was more abundant in the solid tissues normal (Fig. 7, left panel). To note, *Fusobacterium* is particularly present in the cancers related to the gastrointestinal tract (head and neck squamous cell carcinoma, esophageal carcinoma, colon adenocarcinoma and rectum adenocarcinoma). *Mycoplasma* was not as frequently observed as *Fusobacterium*, but still present more in the solid tumor tissues compared to the normal tissues adjacent to the tumor site (Fig. 7, right panel). As observed before, *Mycoplasma* also showed high abundance in the cancer tissues belonging to the gastrointestinal tract. On the other hand, lung squamous cell carcinoma and cholangiocarcinoma presented higher abundance of *Mycoplasmas* in the normal tissues compared to the primary solid tumor (Fig. 7, right panel).

Finally, although overall concordance between the TGCA data set and in vivo findings has been previously reported [2], we decided to further validate our analysis by assessing the presence of both *Mycoplasma* and *Fusobacterium* DnaKs in primary cancer tissues samples of both stomach and colon adenocarcinoma. Both bacteria were readily detected by quantitative real-time PCR with specific primers and probe, in both tumor tissues in variable amount (Additional file 4: Table S1).

## Discussion

**Fig. 8:**
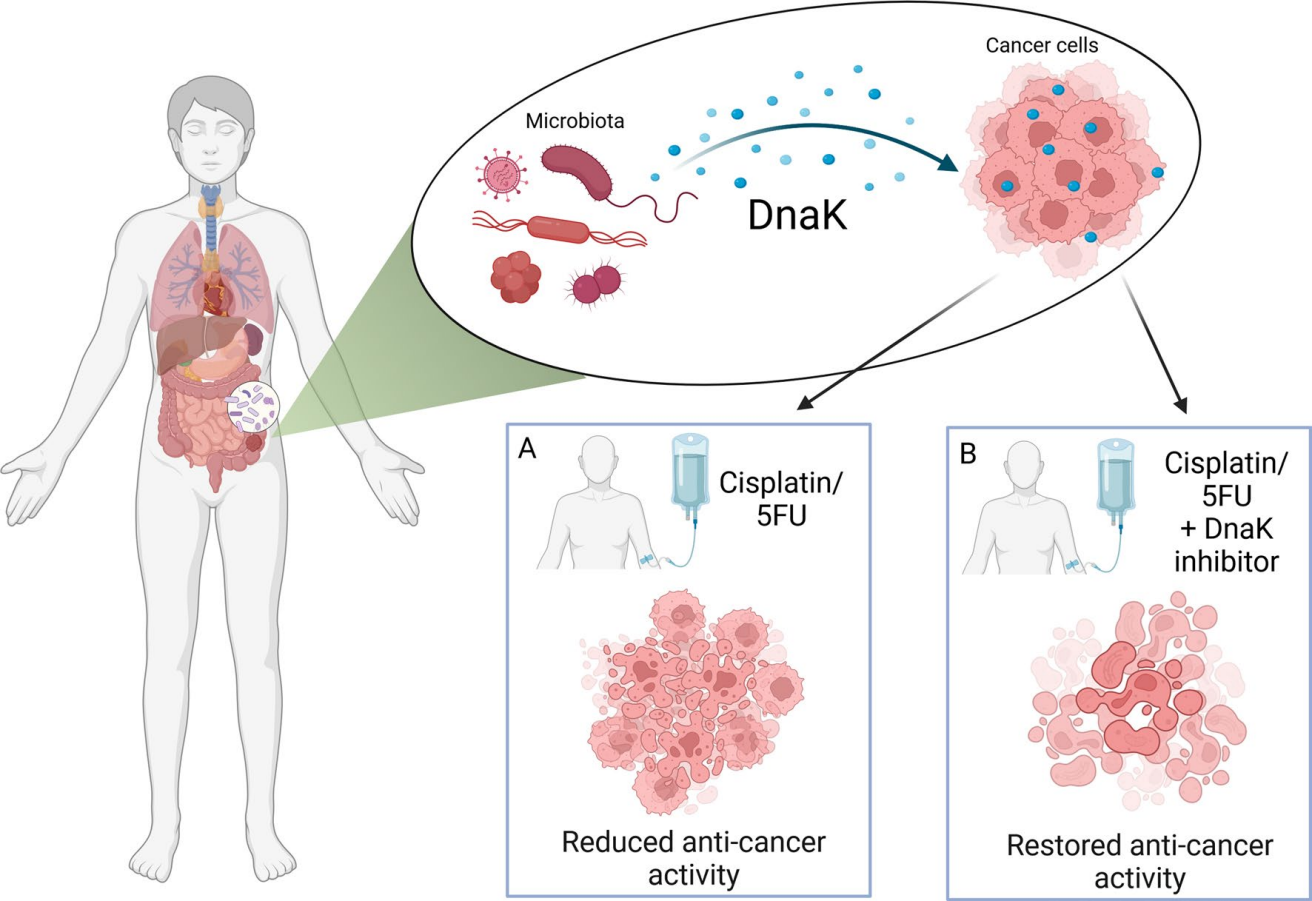
Graphical representation depicting the inhibitory effect of DnaK on anti-cancer activity of Cisplatin and 5FU. **A** Treatment in the presence of DnaK results in reduced activity of anticancer drugs. **B** Adding an inhibitor of DnaK ATP-ase activity restores the activity of the anti-cancer drugs. The figure has been created with BioRender.com

Here we show that *Mycoplasma* DnaK inhibits the anti-cancer effects of widely used anti-cancer drugs (cisplatin and 5FU) in HCT116 and AGS, colorectal and gastric carcinoma cell lines, respectively. We also show that a DnaK binding peptide (ARV-1502) can fully reverse this inhibitory effect. These data were confirmed in a spontaneous murine primary tumor from a knock-in mouse model constitutively expressing *M. fermentans* DnaK generated in our laboratory. Additional studies are ongoing to determine the exact molecular mechanisms responsible for this effect. Subsequently, by analyzing and comparing the distribution of bacteria in human cancer sequencing data sets obtained from TCGA, we identified several other CAB with DnaK highly similar to *Mycoplasma* DnaK in amino acid composition, suggesting their involvement in reducing the efficacy of chemotherapy. Among them, we identified *F. nucleatum*, and we provide evidence that also its DnaK reduces both cisplatin and 5FU anti-cancer activity, in turn restored by ARV-1502 (Fig. 8).

The use in this study of data from TCGA provides an additional layer of clinical relevance to our findings and further help to elucidate the role of specific components of the human cancer microbiota, namely *Mycoplasma* and *Fusobacterium*. We note that these bacteria were previously shown to reduce the anti-cancer activity of drugs like cisplatin and/or 5FU both in vitro and in vivo [8, 10, 12, 13]. Furthermore, recent research revealed that in cancer patients undergoing cisplatin and 5FU treatment, the level of *F. nucleatum* was a predictive marker for chemotherapy response, although the exact molecular mechanism(s) behind this observation was not investigated [11]. It tempting to speculate that increased levels of bacteria are correlated to increased levels of DnaK expression. Additional studies are needed to correlate these levels with response to therapy.

Based on our data, we propose that DnaK reaches the intracellular compartments by two routes: (i) taken up by cancer cells [30, 31, 36] after being expressed and secreted by bacteria present in the tumor microenvironment, and (ii) by being expressed and secreted inside tumor cells by invading bacteria like *Mycoplasmas* [39, 62] or *Fusobacteria* [63]. Intracellular DnaK then binds and reduces the activity of host proteins (such as p53) involved in the effective response to certain anti-cancer drugs [30, 31, 39]. DnaK interaction with co-chaperone proteins, including the co-chaperone DnaJ [30], could provide the necessary ATPase activity for the chaperone function inside the eukaryotic cell [64]. Since ARV-1502 binds the DnaK ATP-ase region and inhibits the ATP-ase activity [43, 44], the peptide would likely act by “locking” DnaK in a conformation unable to bind and inhibit the client proteins’ functions, thus restoring the drugs’ anti-cancer activity.

Given their capacity to interact with several crucial proteins, DnaKs have the potential to influence significant cellular pathways and functions in healthy cells [65]. Notably, their engagement with DNA repair components can render cells more susceptible to transformation following damage, a phenomenon we have recently demonstrated in vivo [66]. Moreover, the inappropriate activation of protein kinases due to DnaK interaction may lead to abnormal cellular activation [42]. We have also found that the presence of DnaK is associated with an increase in Reactive Oxygen Species and pro-inflammatory cytokine production, which may contribute to cancer onset and progression [66]. Highlighting the functional parallels between DnaK and the HSP70 protein family, it's important to note that the HSP70 family is overexpressed in various cancers, where they facilitate growth and survival of cancer cells [67, 68]. This connection not only emphasizes DnaK’s critical contributions to cancer biology but also reinforces its importance as a focal point in cancer research.

## Conclusions

In conclusion, the significant finding of our study is that two CAB, *M. fermentans* and *F. nucleatum*, use a novel mechanism to reduce the efficacy of anti-cancer drugs. Moreover, the use of TCGA data provides a broader context for the clinical significance of these findings. Finally, this discovery offers a practical framework for designing and implementing novel personalized anti-cancer strategies by targeting specific bacterial DnaKs in patients with poor response to chemotherapy.

### Supplementary Information

**Additional file 1: Figure S1.** **A** Direct binding of eM-DnaK to ARV-1502 as determined by surface plasmon resonance (SPR). Association of ARV-1502 at different concentrations on 2274.9 response units of eM-DnaK immobilized on a CM5 biosensor chip proceeded at a flow rate of 35 μL/min for 250 s, followed by a 600 s dissociation in HBS-EP. A preliminary kinetic analysis yielded a Kd value of 1.899e^−6^M. **B** ARV-1502 binds to eM-DnaK and does not prevent eM-DnaK entry into HCT116 cells. eM-DnaK was incubated for 3 h with ARV-1502 and then added to HCT116 cells. After 24 h of incubation cells were treated for 48 h with cisplatin. Western blotting analysis shows that eM-DnaK is able to enter into HCT116 cell line despite the binding of ARV-1502 to DnaK and the treatment with cisplatin. Cells not treated with eM-DnaK, ARV-1502 and cisplatin were used as control.

**Additional file 2: Figure S2.** Top panel—Total number of reads sequenced per cancer type and tissue type (primary solid tumor and solid tissue normal). Bottom panel—Number of 16S sequences per cancer type and tissue type (primary solid tumor and solid tissue normal). The total number of reads was determined in the different cancer samples belonging to the TCGA data set, as described in “Materials and methods”.

**Additional file 3: Figure S3.** **A** Distribution of the number of samples across cancer types retrieved from TCGA (n = 10,293). **B** Post-filtering distribution of the primary solid tumor and solid tissue normal samples across cancer types included in the analyses.

**Additional file 4: Table S1.** qPCR shows variable copy number of Mycoplasma and Fusobacterium DnaK in primary cells from colon and stomach cancers. (ND: not detected).

**Additional file 5: Table S2.** Template sequence used for DnaK domains’ comparison.

## Data Availability

The data generated in this study are available within the article and its additional files.

## Abbreviations

CAB: Cancer-associated bacteria
*M. fermentans*: *Mycoplasma fermentans*
*F. nucleatum*: *Fusobacterium nucleatum*
eM-DnaK: Exogenous *Mycoplasma fermentans* DnaK
eF-DnaK: Exogenous *Fusobacterium nucleatum* DnaK
5FU: 5-Fluorouracil
TCGA: The Cancer Genome Atlas
Hsp70: 70-KDa heat shock protein
USP10: Ubiquitin carboxyl-terminal hydrolase 10
SPR: Surface plasmon resonance
