# Supplementary material for: Bacterial DnaK reduces the activity of anti-cancer drugs cisplatin and 5FU
Source: J Transl Med. 2024 Mar 12;22:269. doi: 10.1186/s12967-024-05078-x (PMC10935962; doi:10.1186/s12967-024-05078-x)
Supplement: Supplementary file 1 — Additional file 1: Figure S1. A Direct binding of eM-DnaK to ARV-1502 as determined by surface plasmon resonance (SPR). Association of ARV-1502 at different concentrations on 2274.9 response units of eM-DnaK immobilized on a CM5 biosensor chip proceeded at a flow rate of 35 μL/min for 250 s, followed by a 600 s dissociation in HBS-EP. A preliminary kinetic analysis yielded a Kd value of 1.899e−6M. B ARV-1502 binds to eM-DnaK and does not prevent eM-DnaK entry into HCT116 cells. eM-DnaK was incubated for 3 h with ARV-1502 and then added to HCT116 cells. After 24 h of incubation cells were treated for 48 h with cisplatin. Western blotting analysis shows that eM-DnaK is able to enter into HCT116 cell line despite the binding of ARV-1502 to DnaK and the treatment with cisplatin. Cells not treated with eM-DnaK, ARV-1502 and cisplatin were used as control. [file 12967_2024_5078_MOESM1_ESM.pdf]

Fig.S1

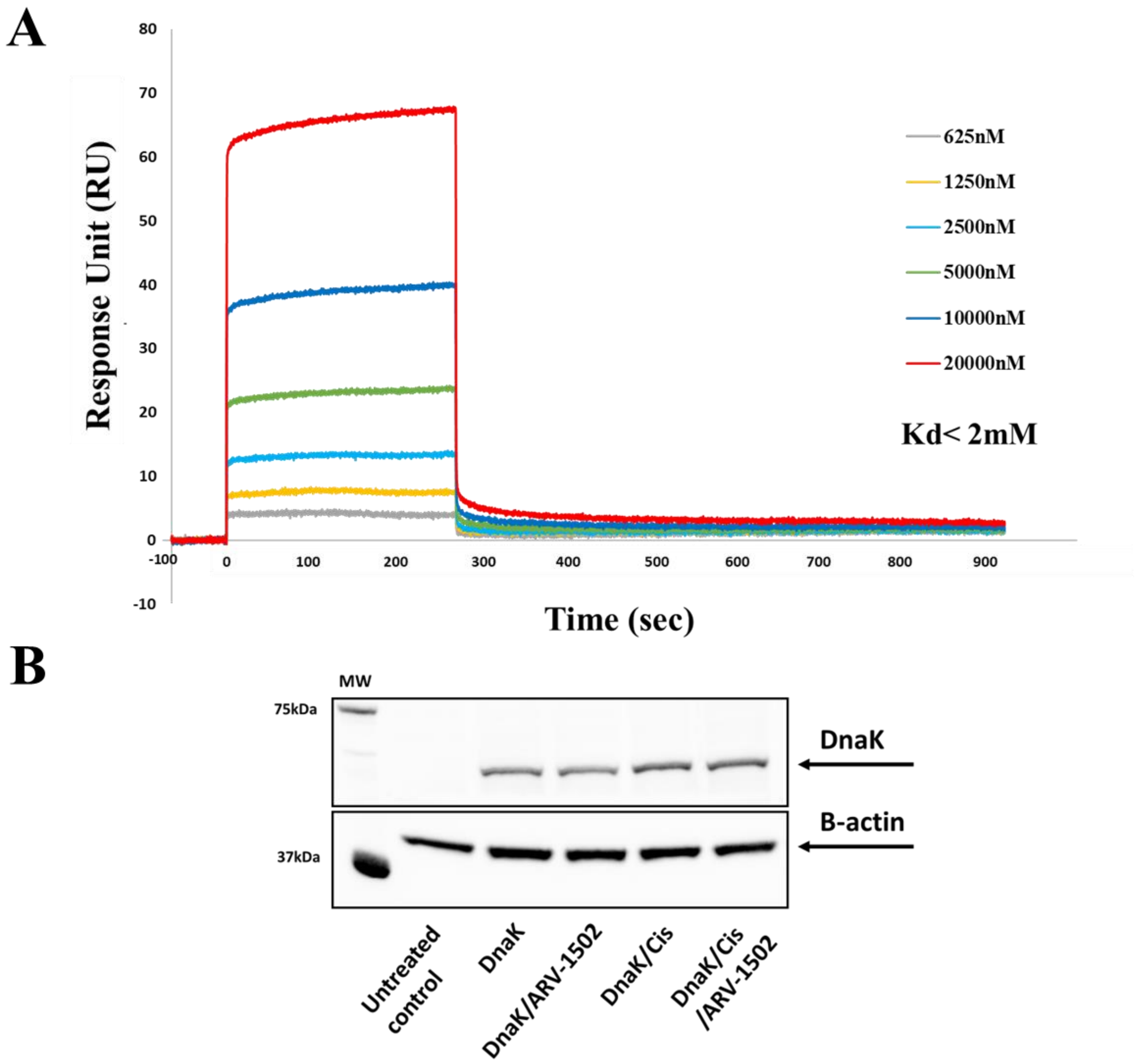
A
Response Unit (RU)
80
70
60
50
40
30
20
10
0
-10
-100
0
100
200
300
400
500
600
700
800
900
Time (sec)
625nM
1250nM
2500nM
5000nM
10000nM
20000nM
Kd< 2mM
B
MW
75kDa
37kDa
DnaK
B-actin
Untreated control
DnaK
DnaK/ARV-1502
DnaK/Cis
DnaK/Cis /ARV-1502

Fig.S1 A) Direct binding of eM-DnaK to ARV-1502 as determined by surface plasmon resonance (SPR). Association of ARV-1502 at different concentrations on 2274.9 response units of eM-DnaK immobilized on a CM5 biosensor chip proceeded at a flow rate of 35 µl/min for 250 sec, followed by a 600 sec dissociation in HBS-EP. A preliminary kinetic analysis yielded a Kd value of $1.899e^{-6}$M. **B) ARV-1502 binds to eM-DnaK and does not prevent eM-DnaK entry into HCT116 cells.** eM-DnaK was incubated for 3 hours with ARV-1502 and then added to HCT116 cells. After 24 hours of incubation cells were treated for 48 hours with cisplatin. Western blotting analysis shows that eM-DnaK is able to enter into HCT116 cell line despite the binding of ARV-1502 to DnaK and the treatment with cisplatin. Cells not treated with eM-DnaK, ARV-1502 and cisplatin were used as control.
