# Supplementary material for: Bacterial DnaK reduces the activity of anti-cancer drugs cisplatin and 5FU
Source: J Transl Med. 2024 Mar 12;22:269. doi: 10.1186/s12967-024-05078-x (PMC10935962; doi:10.1186/s12967-024-05078-x)
Supplement: Supplementary file 2 — Additional file 2: Figure S2. Top panel—Total number of reads sequenced per cancer type and tissue type (primary solid tumor and solid tissue normal). Bottom panel—Number of 16S sequences per cancer type and tissue type (primary solid tumor and solid tissue normal). The total number of reads was determined in the different cancer samples belonging to the TCGA data set, as described in “Materials and methods”. [file 12967_2024_5078_MOESM2_ESM.pdf]

Fig.S2

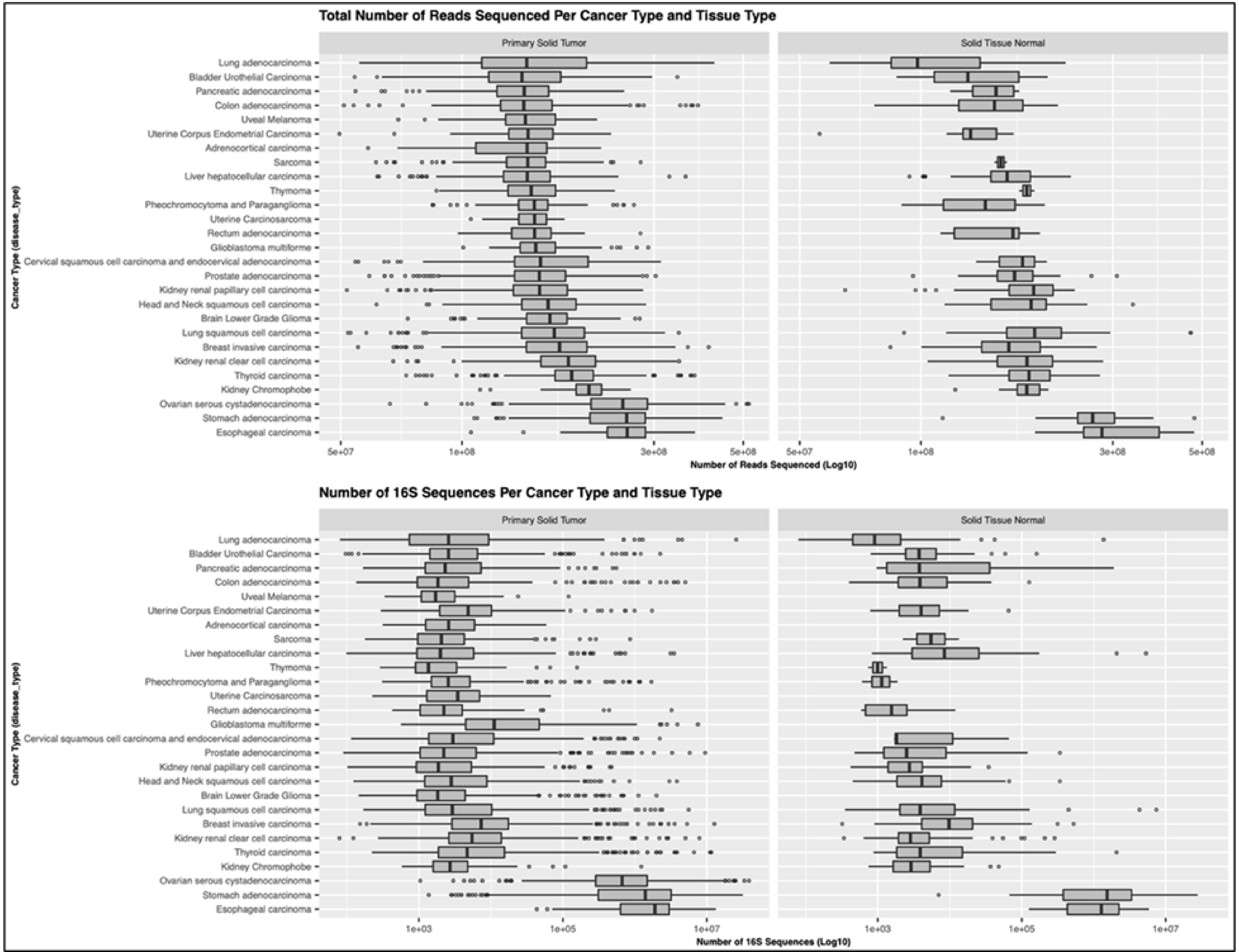
Total Number of Reads Sequenced Per Cancer Type and Tissue Type
Primary Solid Tumor
Solid Tissue Normal
Lung adenocarcinoma
Bladder Urothelial Carcinoma
Pancreatic adenocarcinoma
Colon adenocarcinoma
Uveal Melanoma
Uterine Corpus Endometrial Carcinoma
Adrenocortical carcinoma
Sarcoma
Liver hepatocellular carcinoma
Thymoma
Pheochromocytoma and Paraganglioma
Uterine Carcinosarcoma
Rectum adenocarcinoma
Glioblastoma multiforme
Cervical squamous cell carcinoma and endocervical adenocarcinoma
Prostate adenocarcinoma
Kidney renal papillary cell carcinoma
Head and Neck squamous cell carcinoma
Brain Lower Grade Glioma
Lung squamous cell carcinoma
Breast invasive carcinoma
Kidney renal clear cell carcinoma
Thyroid carcinoma
Kidney Chromophobe
Ovarian serous cystadenocarcinoma
Stomach adenocarcinoma
Esophageal carcinoma
Cancer Type (disease_type)
5e+07
1e+08
3e+08
5e+08
Number of Reads Sequenced (Log10)
Number of 16S Sequences Per Cancer Type and Tissue Type
1e+03
1e+05
1e+07
Number of 16S Sequences (Log10)

Fig.S2 Top panel - Total number of reads sequenced per cancer type and tissue type (primary solid tumor and solid tissue normal). **Bottom panel - Number of 16S sequences per cancer type and tissue type** (primary solid tumor and solid tissue normal). The total number of reads was determined in the different cancer samples belonging to the TCGA data set, as described in Materials and Methods.
