# Supplementary material for: Bacterial DnaK reduces the activity of anti-cancer drugs cisplatin and 5FU
Source: J Transl Med. 2024 Mar 12;22:269. doi: 10.1186/s12967-024-05078-x (PMC10935962; doi:10.1186/s12967-024-05078-x)
Supplement: Supplementary file 3 — Additional file 3: Figure S3. A Distribution of the number of samples across cancer types retrieved from TCGA (n = 10,293). B Post-filtering distribution of the primary solid tumor and solid tissue normal samples across cancer types included in the analyses. [file 12967_2024_5078_MOESM3_ESM.pdf]

Fig.S3A

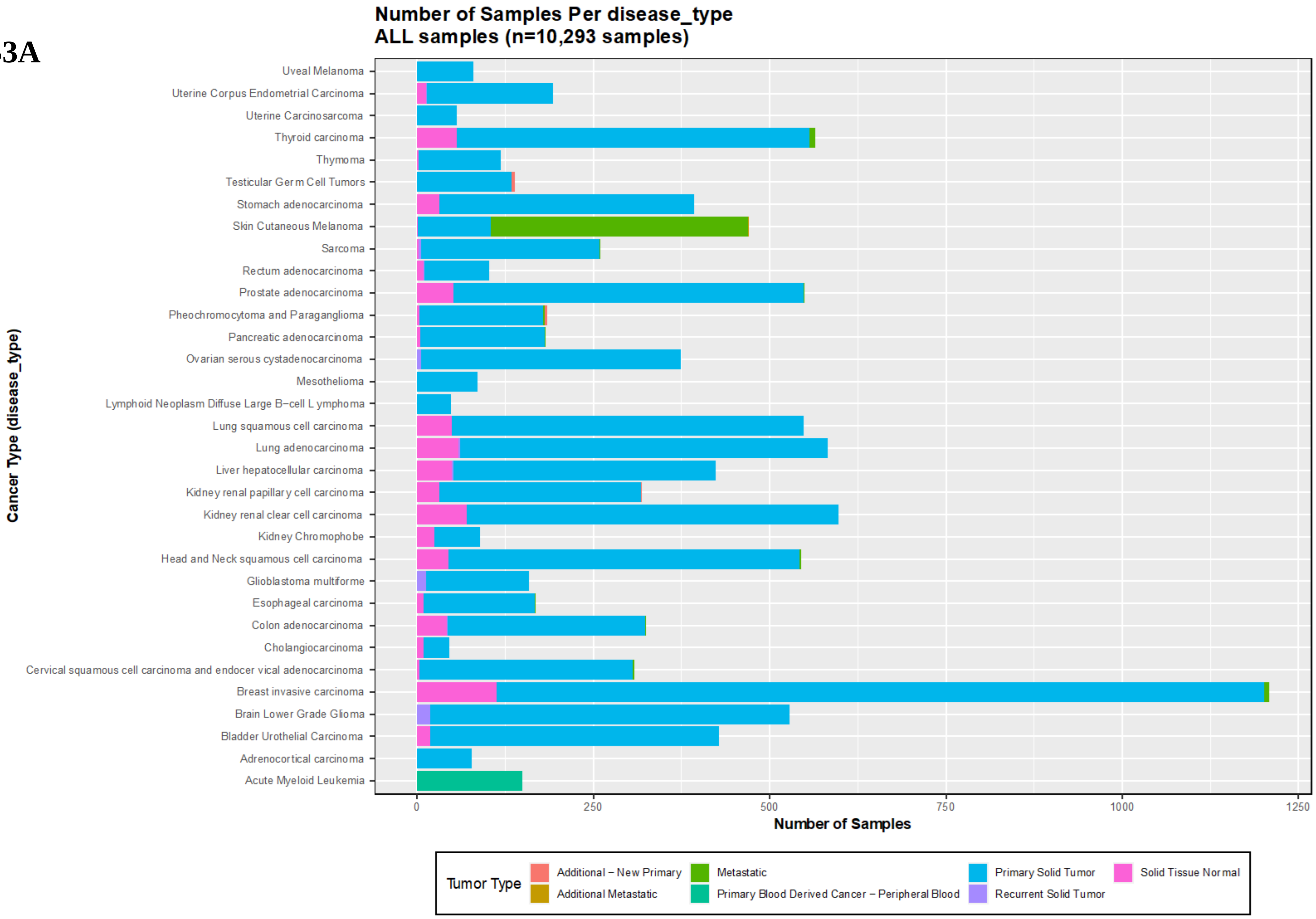

Number of Samples Per disease_type
ALL samples (n=10,293 samples)
Cancer Type (disease_type)
Uveal Melanoma
Uterine Corpus Endometrial Carcinoma
Uterine Carcinosarcoma
Thyroid carcinoma
Thymoma
Testicular Germ Cell Tumors
Stomach adenocarcinoma
Skin Cutaneous Melanoma
Sarcoma
Rectum adenocarcinoma
Prostate adenocarcinoma
Pheochromocytoma and Paraganglioma
Pancreatic adenocarcinoma
Ovarian serous cystadenocarcinoma
Mesothelioma
Lymphoid Neoplasm Diffuse Large B−cell Lymphoma
Lung squamous cell carcinoma
Lung adenocarcinoma
Liver hepatocellular carcinoma
Kidney renal papillary cell carcinoma
Kidney renal clear cell carcinoma
Kidney Chromophobe
Head and Neck squamous cell carcinoma
Glioblastoma multiforme
Esophageal carcinoma
Colon adenocarcinoma
Cholangiocarcinoma
Cervical squamous cell carcinoma and endocervical adenocarcinoma
Breast invasive carcinoma
Brain Lower Grade Glioma
Bladder Urothelial Carcinoma
Adrenocortical carcinoma
Acute Myeloid Leukemia
0
250
500
750
1000
1250
Number of Samples
Tumor Type
Additional − New Primary
Additional Metastatic
Metastatic
Primary Blood Derived Cancer − Peripheral Blood
Primary Solid Tumor
Recurrent Solid Tumor
Solid Tissue Normal

Fig.S3B

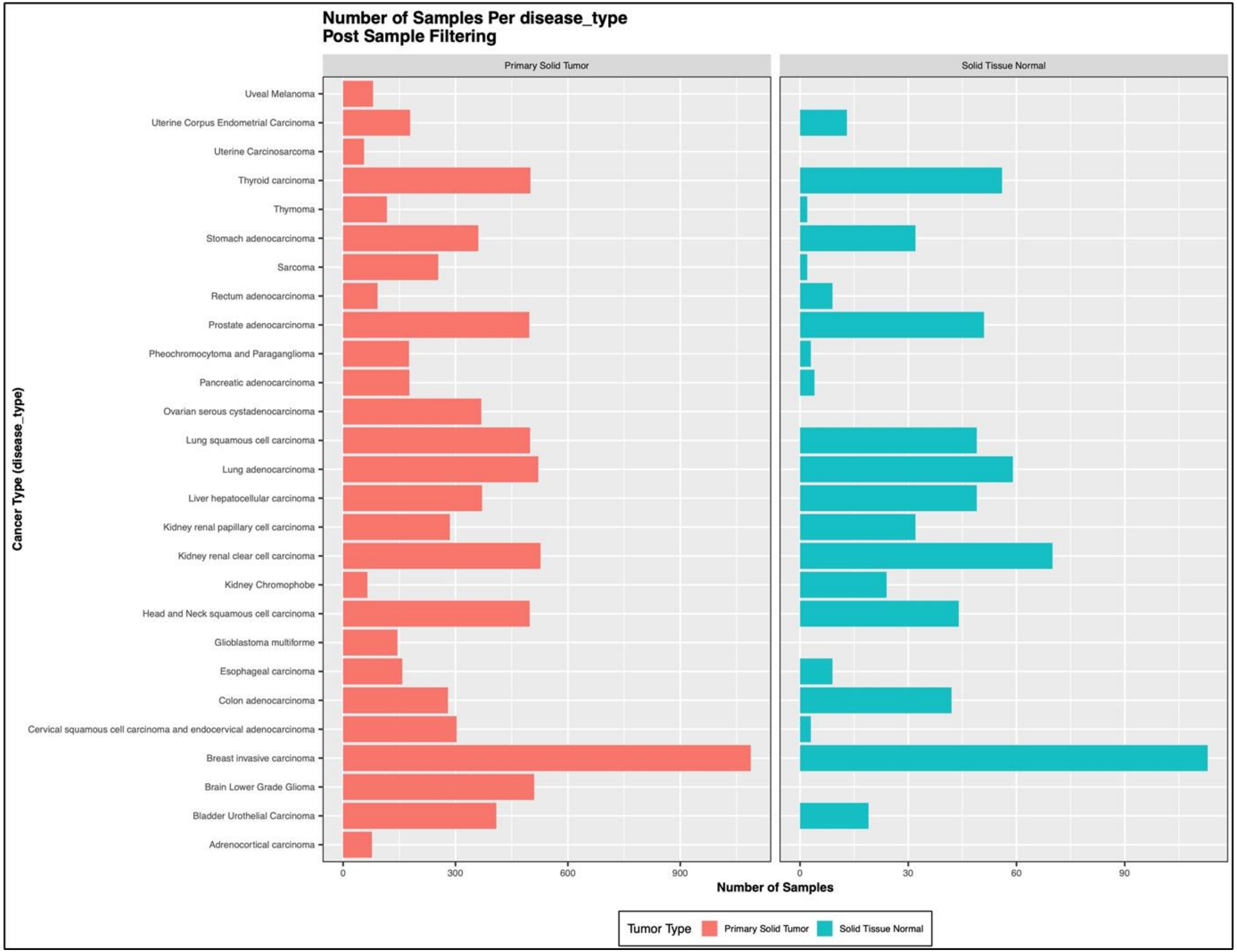
Number of Samples Per disease_type
Post Sample Filtering
Primary Solid Tumor
Solid Tissue Normal
Uveal Melanoma
Uterine Corpus Endometrial Carcinoma
Uterine Carcinosarcoma
Thyroid carcinoma
Thymoma
Stomach adenocarcinoma
Sarcoma
Rectum adenocarcinoma
Prostate adenocarcinoma
Pheochromocytoma and Paraganglioma
Pancreatic adenocarcinoma
Ovarian serous cystadenocarcinoma
Lung squamous cell carcinoma
Lung adenocarcinoma
Liver hepatocellular carcinoma
Kidney renal papillary cell carcinoma
Kidney renal clear cell carcinoma
Kidney Chromophobe
Head and Neck squamous cell carcinoma
Glioblastoma multiforme
Esophageal carcinoma
Colon adenocarcinoma
Cervical squamous cell carcinoma and endocervical adenocarcinoma
Breast invasive carcinoma
Brain Lower Grade Glioma
Bladder Urothelial Carcinoma
Adrenocortical carcinoma
Cancer Type (disease_type)
0
300
600
900
0
30
60
90
Number of Samples
Tumor Type
Primary Solid Tumor
Solid Tissue Normal

Fig.S3 A) Distribution of the number of samples across cancer types retrieved from TCGA (n=10,293). B) Post- filtering distribution of the primary solid tumor and solid tissue normal samples across cancer types included in the analyses.
