# Supplementary material for: Bacterial DnaK reduces the activity of anti-cancer drugs cisplatin and 5FU
Source: J Transl Med. 2024 Mar 12;22:269. doi: 10.1186/s12967-024-05078-x (PMC10935962; doi:10.1186/s12967-024-05078-x)
Supplement: Supplementary file 4 — Additional file 4: Table S1. qPCR shows variable copy number of Mycoplasma and Fusobacterium DnaK in primary cells from colon and stomach cancers. (ND: not detected). [file 12967_2024_5078_MOESM4_ESM.pdf]

Table S1. qPCR shows variable copy number of Mycoplasma and Fusobacterium DnaK in primary cells from colon and stomach cancers. (ND: not detected)

Patient ID	Tumor Site	M-DnaK DNA copies/50ng total DNA	F-DnaK DNA copies/50ng total DNA
30284	Colon	95100	4800
30357	Colon	10000	1000
P0859	Colon	3720	2580
30549	Colon	ND	3280
P0860	Colon	3640	ND
30304	Stomach	15940	1140
P0861	Stomach	296560	2880
P0862	Stomach	3400	860
30404	Stomach	81560	15760
