# Supplementary material for: Bacterial DnaK reduces the activity of anti-cancer drugs cisplatin and 5FU
Source: J Transl Med. 2024 Mar 12;22:269. doi: 10.1186/s12967-024-05078-x (PMC10935962; doi:10.1186/s12967-024-05078-x)
Supplement: Supplementary file 5 — Additional file 5: Table S2. Template sequence used for DnaK domains’ comparison. [file 12967_2024_5078_MOESM5_ESM.pdf]

Table S2. Template sequence used for DnaK domains' comparison.

```
>Mycoplasma fermentans no info;1-366;367-481;482-585
MAKETIIGIDLGTTNSAVAIVDGGTPIVLENYNGKRTTPSVVSFKDGEIIVGENAKNQIETNPDTIASVKRFM
GTKKIFKANGKEYKPEEISAIILDHLRKYAEEKVGHKIEKAVITVPAYFDNAQREATKIAGKIAGLDVLRIIN
EPTAAALAFGLDKTNKEMKVLVFDLGGGTFDVSILELADGTFEVLATSGDNKLGGDDWDHEIVDWLVAKIKND
HKIDIRENKMAMARLKAAAEKAKIDLSSSLVAHISLPFLVLLDNHEPINVEAELKRSEFEKMTAKLVERCRRP
IQDALSEAKLKISDLDEILLVGGSTRIPAVQALVEKILNRKPNKSVNPDEVVAMGAAIQGAVLAGDINDILLV
DVTPLTLGIETAGGISTPLIPRNTRIPITKSETFTTFENNQTDVTIKIVQGERPVASENKLLGQFNLTGIRPA
PRGIPQIEVSFKIDANGITTVSAKDKDTQKEQSITIKNSSKLSEEEVERMIKEAEENREADAKRAADIEIIVR
AETMVAKFESVLEENKDKLTQDQINQAQAEIDKINGFIKEKEYDQLRLTIKAFEELLDSMSNADSSSFKEEDA
E
```
